# Photobiomodulation by Near-Infrared 980-nm Wavelengths Regulates Pre-Osteoblast Proliferation and Viability through the PI3K/Akt/Bcl-2 Pathway

**DOI:** 10.3390/ijms22147586

**Published:** 2021-07-15

**Authors:** Dimitrios Agas, Reem Hanna, Stefano Benedicenti, Nicola De Angelis, Maria Giovanna Sabbieti, Andrea Amaroli

**Affiliations:** 1 School of Biosciences and Veterinary Medicine, University of Camerino, Camerino, 62032 Macerata, Italy; dimitrios.agas@unicam.it (D.A.); giovanna.sabbieti@unicam.it (M.G.S.); 2 Department of Oral Surgery, Dental Institute, King’s College Hospital NHS Foundation Trust, Denmark Hill, London SE5 9RS, UK; reemhanna@hotmail.com; 3 Department of Surgical and Diagnostic Sciences, University of Genoa, 16132 Genoa, Italy; stefano.benedicenti@unige.it (S.B.); n.deangelis74@gmail.com (N.D.A.); 4 Department of Orthopaedic Dentistry, First Moscow State Medical University (Sechenov University), 11991 Moscow, Russia

**Keywords:** photobiomodulation, low-level laser therapy, light therapy, photo-therapy, bone regeneration, bone differentiation, apoptosis, proliferation, osteoblast, mechanotransduction

## Abstract

Background: bone tissue regeneration remains a current challenge. A growing body of evidence shows that mitochondrial dysfunction impairs osteogenesis and that this organelle may be the target for new therapeutic options. Current literature illustrates that red and near-infrared light can affect the key cellular pathways of all life forms through interactions with photoacceptors within the cells’ mitochondria. The current study aims to provide an understanding of the mechanisms by which photobiomodulation (PBM) by 900-nm wavelengths can induce in vitro molecular changes in pre-osteoblasts. Methods: The PubMed, Scopus, Cochrane, and Scholar databases were used. The manuscripts included in the narrative review were selected according to inclusion and exclusion criteria. The new experimental set-up was based on irradiation with a 980-nm laser and a hand-piece with a standard Gaussian and flat-top beam profile. MC3T3-E1 pre-osteoblasts were irradiated at 0.75, 0.45, and 0.20 W in continuous-wave emission mode for 60 s (spot-size 1 cm^2^) and allowed to generate a power density of 0.75, 0.45, and 0.20 W/cm^2^ and a fluence of 45, 27, and 12 J/cm^2^, respectively. The frequency of irradiation was once, three times (alternate days), or five times (every day) per week for two consecutive weeks. Differentiation, proliferation, and cell viability and their markers were investigated by immunoblotting, immunolabelling, fluorescein-FragELTM-DNA, Hoechst staining, and metabolic activity assays. Results and conclusions: The 980-nm wavelength can photobiomodulate the pre-osteoblasts, regulating their metabolic schedule. The cellular signal activated by 45 J/cm^2^, 0.75 W and 0.75 W/cm^2^ consist of the PI3K/Akt/Bcl-2 pathway; differentiation markers were not affected, nor do other parameters seem to stimulate the cells. Our previous and present data consistently support the window effect of 980 nm, which has also been described in extracted mitochondria, through activation of signalling PI3K/Akt/Bcl-2 and cyclin family, while the Wnt and Smads 2/3-β-catenin pathway was induced by 55 J/cm^2^, 0.9 W and 0.9 W/cm^2^.

## 1. Introduction

The percentage of unhealed bone fracture complications in humans is notable. Indeed, in the United States of America, about 50 million annual tooth extractions and a considerable number of apicectomies leave bone defects. The postoperative complications of these procedures are quantified in at least 30,000 patients/year worldwide [1,2,3]. In previous work, we evidenced that bone tissue regeneration following oral and maxillofacial surgery remains a current challenge in dentistry [3].

More than 10% of oral-bone surgeries and related procedures can have healing complications due to either infection, severe tissue damage, large bone defects, or compromised blood supply [4], which appears to be a key pathophysiological event in both slow recovery and lack of healing [5]. Indeed, lack of oxygen delivery to the cells due to blood supply deprivation has a significant impact on the cellular production of energy for the tissue during the healing process (from protein synthesis to cell migration and neovascularization) [4,5,6,7,8,9]. A growing body of evidence has demonstrated that mitochondrial dysfunction impairs osteogenesis [10] and that this organelle may be a target for new therapeutic options to treat metabolic bone diseases [11]. It has been demonstrated that direct delivery of adenosine triphosphate (ATP) to the cells improves tissue healing in rabbit [7] and mouse [8] models. This fact suggests that the major barrier towards effective biological healing is insufficient energy.

Up-to-date literature shows that red and near-infrared (NIR) light can affect the key cellular pathways of all life forms by interacting with specific photoacceptors within the cell [12]. The resulting medical topic is commonly known as low-level laser/light therapy (LLLT), but the more appropriate term photobiomodulation (PBM) therapy was recently affirmed. The reliability of the photobiomodulatory event is strongly based on biological and physical-chemical evidence [13,14].

From a cellular point of view, the best-studied wavelengths for bone regeneration are 600 nm, showing that, in accordance with specific irradiation parameters, pre-osteoblast proliferation and viability can either be positively affected [15,16,17,18,19,20,21,22,23] or not influenced [24,25,26,27,28,29] by PBM therapy. Pre-osteoblast differentiation/homeostasis is strongly modulated by 630–685 nm range light. In fact, there has been a debate between authors that showed increased activity of both differentiation markers and matrix mineralisation [17,20,22,23,25,28], and others that found no influence after laser irradiation treatments [19,24,26].

Similarly, PBM at the 800-nm wavelengths has been shown to affect both proliferation and viability of pre-osteoblasts either positively [23,27,30,31,32,33] or negatively [27,28]; however, in some cases, cells were not influenced [34,35]. Pre-osteoblast differentiation is likewise modulated by 800-nm range PBM, but various authors showed an increase [23,29,30,33,36,37,38] or decrease [28] in differentiation markers, or no response [34,35].

Concerning the wavelengths in the range of 905–980 nm, careful screening of the PubMed, Scopus, and Cochrane databases showed that they have not been widely investigated compared to the lower wavelengths. This is also supported by a thought-provoking recent review [39].

It should, however, be emphasised that the term PBM describes the event of light interaction modulating cellular metabolism, but the players involved may change as the wavelength moves from visible light, through NIR (780–890 nm), up to the 905–1064 nm range, as we recently discussed [13,14]. Cytochromes, bound-water, voltage-dependent receptors, lipids, and nitrosothiol (thionitrite) compounds are able to function as photoacceptors, but do so in selective modalities with respect to the wavelengths involved [13].

Hence, the current study aims to provide a comprehensive understanding of the mechanisms by which PBM therapy in the 905–980 nm range of wavelengths can induce in vitro molecular changes in bone regeneration. Specifically, we discuss the literature concerning the effect of 900-nm wavelengths on osteoblast cell lines and, according to our previous results [14,40], we evaluate the effects of 980-nm laser light irradiation on MC3T3-E1 pre-osteoblast proliferation and differentiation at various irradiation parameter settings. Moreover, we compare the effectiveness of an innovative technology that allows irradiation mediated by a hand-piece showing a flat-top (FT) beam profile versus the standard, Gaussian (ST) beam profile.

## 2. Results

### 2.1. Effect of Photobiomodulation on Temperature

There was no statistically significant difference in measures of the medium and monolayer temperature before and after the laser irradiation (data not shown).

### 2.2. Effect of Photobiomodulation on the Viability and Proliferation of Pre-Osteoblasts

**Figure 1:**
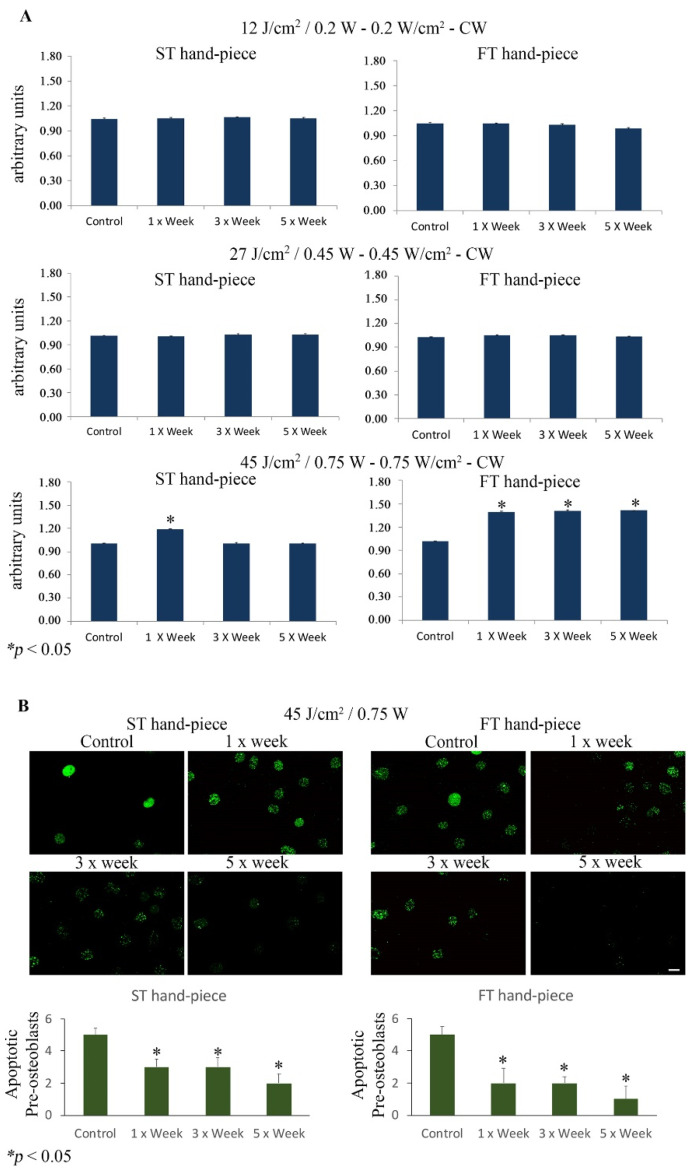
(**A**). Effect of photobiomodulation on pre-osteoblast metabolic activity. The MTS assay (*n* = 6) was performed after the MC3T3-E1 cell line was irradiated by 12 J/cm^2^/0.20 W, 27 J/cm^2^/0.45 W, or 45 J/cm^2^/0.75 W using the standard (ST) or flat-top (FT) hand-piece. The frequency of irradiation was once per week (1× Week), three times (alternate day, 3× Week), and five times (every day, 5× Week). Note the increased cell viability after the 45 J/cm^2^/0.75 W treatment with the ST hand-piece at 1× per week and with the FT hand-piece at all time point protocols (values represent means ± S.E., *p* < 0.05 compared to the control). (**B**). Representative photomicrographs of Fluorescein-FragEL-positive osteoblasts that decreased after ST hand-piece and FT hand-piece laser treatment at 45 J/cm^2^/0.75 W for 1× week until 5× week. The quantitative analysis was performed as previously described in Materials and Methods Section; * *p* < 0.05. Bar, 10 µm.

The results showed a statistically significant increase in cell viability (*p* < 0.05) in the group irradiated with the ST hand-piece once a week and with 45 J/cm^2^, 0.75 W. However, it has to be noted that the FT hand-piece induced a statistically significant increase (*p* < 0.05) in the cell viability in the cultures irradiated once, three, and five times per week. Moreover, no effects were observed in the ST and FT hand-piece lasered samples (*p* > 0.05) when irradiated with 12 J/cm^2^, 0.20 W or 27 J/cm^2^, 0.45 W. Where induced, the effect was significantly higher in the samples irradiated with the FT beam profile than the ST beam profile (Figure 1A).

Notably, the fluorescein FragELTM assay demonstrated a statistically significant decrease in apoptotic MC3T3-E1 osteoblasts after ST hand-piece and FT hand-piece laser treatment at 45 J/cm^2^, 0.75 W at all experimental time points (Figure 1B).

**Figure 2:**
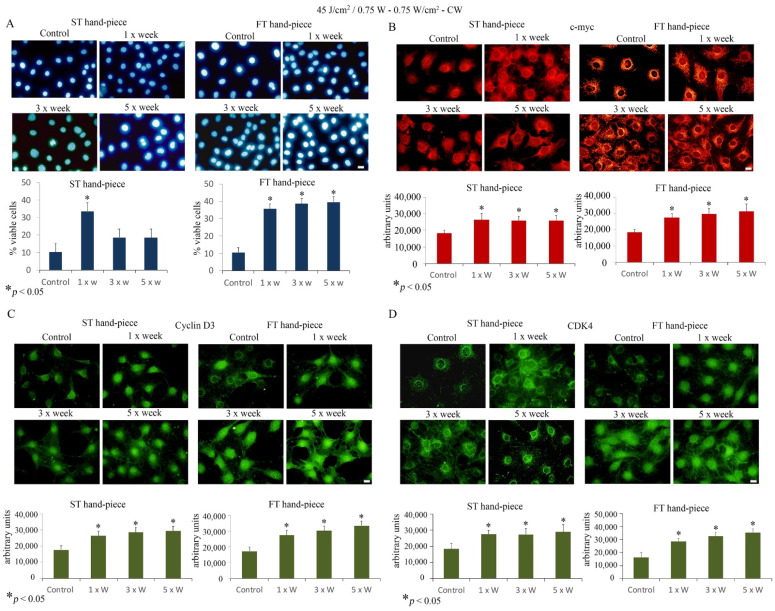
Effect of 45 J/cm^2^–0.75 W laser treatment on pre-osteoblast proliferation. The frequency of irradiation was once per week (1× Week), three times (alternate day, 3× Week), and five times (every day, 5× Week). (**A**). The Hoechst stain assay revealed increased cell viability using both standard (ST) and flat-top (FT) hand-pieces. Notably, the FT device has managed to yet again increase the efficacy of the laser treatment at all time points applied (values represent means ± S.E., *p* < 0.05 compared to the control). (**B**–**D**). Single immunolabeling for c-myc (**B**) cyclin D3 (**C**) and CDK4 (**D**). The Fluorescence analysis was quantified by a Tecan Infinite fluorescence reader and values were analyzed by Magellan v4.0 software. Asterisks show the statistically significant differences of the samples analyzed compared with the control counterpart. Values represent means ± S.E.; * *p* < 0.05 (*n* = 4). Bar, 10 µm.

Furthermore, Hoechst staining showed an increase in the cell viability after 1× week ST hand-piece treatment and at all time points when the FT hand-piece was applied (Figure 2).

Thus, we questioned whether laser treatment at 45 J/cm^2^, 0.75 W can influence important proliferative markers such as c-myc, CDK4, and cyclin D3. Immunolabeling findings revealed that laser treatment significantly increased c-myc, CDK4, and cyclin D3 protein labelling, which displayed a strong increase, particularly evident in FT hand-piece-treated pre-osteoblast cultures.

### 2.3. Effect of Photobiomodulation on Pre-Osteoblasts Antiapoptotic Signalling Cascades

**Figure 3:**
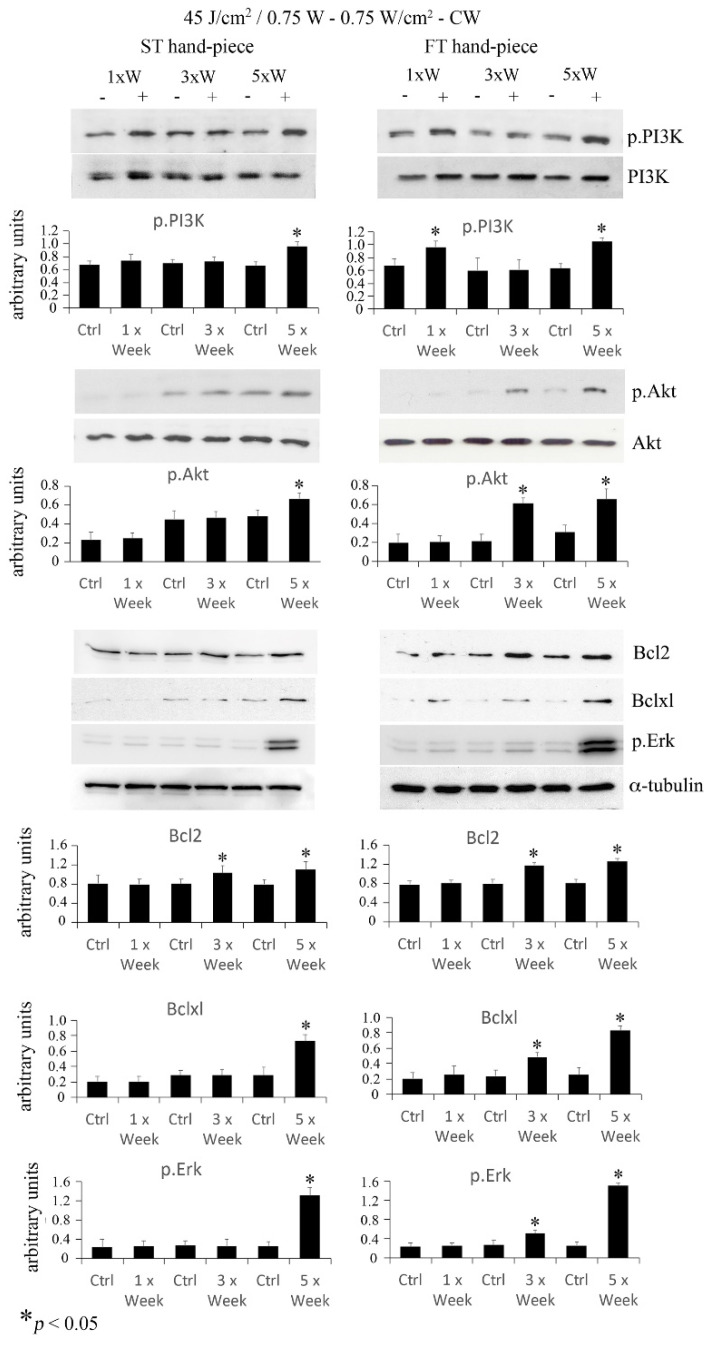
Effect of 45 J/cm^2^–0.75 W laser treatment on anti-apoptotic/pro-survival markers. The frequency of irradiation was once per week (1× Week), three times (alternate day, 3× Week), and five times (every day, 5× Week). Western blotting analysis of p.PI3K, p.Akt, Bcl2, Bclxl, and p.Erk was performed (*n* = 3). Note the increase in pro-survival proteins, which was particularly evident using the FT device (values represent means ± S.E., * *p* < 0.05 compared to the control).

Akt is a serine/threonine-protein kinase that participates in pro-survival/differentiation signal cascades and is activated by phosphatidyl-inositol 3 kinase (PI3K) phosphorylation. To investigate Akt activation, pre-osteoblasts irradiated at a power of 0.75 W and fluence of 45 J/cm^2^. Western blotting analysis revealed that phospho-PI3K increased with the 3× per week and 5× per week irradiation protocols delivered with the ST hand-piece, and at all time points when the FT hand-piece was used. Moreover, an increase in phospho-Akt was found in cultures irradiated 5× per week with the ST hand-piece, and 3× per week and 5× per week with the FT hand-piece. Notably, the applied PBM protocol was able to activate phospho-PI3K and phospho-Akt, which are crucial indicators of pro-survival cell activities. Then, we investigated the downstream targets of the aforementioned signalling hubs, such as the anti-apoptotic Bcl-2 and Bxl-xl proteins and the multifunctional kinase phospho-ERK. As expected, an increase in Bcl-2 expression after 3× per week and 5× per week irradiation was found when pre-osteoblasts were treated with the ST beam profile. The same results were detected in pre-osteoblasts treated with the FT beam profile. Moreover, Bcl-xl was augmented in the pre-osteoblasts when treated with the 5× per week protocol by the ST beam profile and with the 3× per week and 5× per week protocols when treated with the FT beam profile. Finally, phospho-ERK was found to be strongly expressed, with the same trend described for the anti-apoptotic protein Bcl-xl (Figure 3). The obtained results suggested, for the first time, that the PBM protocol using 45 J/cm^2^, 0.75 W, 0.75 W/cm^2^–CW, induces the survival mode of the pre-osteoblasts metabolic schedule.

### 2.4. Effects of Photobiomodulation on Pre-Osteoblast Differentiation

Neither the histochemical nor the immunoblot analyses were able to assess statistically valid results concerning the effects of the irradiation modalities and the administered powers on MC3T3-E1 differentiation (Runx2 and osterix protein blots and alkaline phosphatase assay, data not shown).

### 2.5. Literature Analysis

Only one [40] out of 4796 articles selected from the PubMed, Scopus, Cochrane, and Scholar databases complied with the more selective inclusion criteria (Supplemental Material, Figure S1). Papers were mostly rejected due to missing measurements of both the power-meter and temperature detector. However, when these two criteria were removed, it was possible to include 12 papers in the narrative review (Figure S1).

Following our previous reviews on PBM [3,13,41], the second most frequent reason for exclusion was the unsuitability of the parameters’ reproducibility, as described by Tunér and Jenkins [42]. The 12 selected papers employed the following wavelengths: one article used 905 nm [43], two used 910 nm [44,45] and 980 nm [40,46], three used 915 nm [47,48,49], and four used 940 nm [50,51,52,53]. According to the parameters and mode of irradiation recorded in Table S1, the PBM positively affected the proliferation and cell viability in some studies [40,43,46,48,50,52], despite much evidence supporting no effect [44,46,47,48,49,52]. The authors described a decrease in cell viability after PBM therapy [44,48,52]. The cellular pathways involved in pre-osteoblast differentiation were also modulated by PBM, as pointed out by some authors [40,43,44,45,46,47,48,49,50,51,52,53]. The effect of PBM in the 905–980 nm range of wavelengths is shown in Figure S2 and discussed more thoroughly in the discussion section below.

## 3. Discussion

Photobiomodulation events are the result of intramural cell signalling cascades, with the start point represented by the photoacceptor, which activates second messengers and culminates with the modulation of tissue homeostasis. As previously discussed [3,13,41,54] and also displayed in Figure S2, the primary target can be identified in the cytochromes of the mitochondrial respiratory chain, nitrosothiol compounds, bound water, and lipids. After the interaction with red and particularly infrared light, targets modify their energetic and vibrational state, supporting the release of ATP, ROS, nitric oxide, and calcium through the opening of voltage-dependent receptors and release from intra-organellar-sequestered reserves. It has been clarified that different wavelengths interact with photoacceptors differently, according to their coefficient of absorption. From 450 nm to >900 nm, the affinity of the cytochrome-c oxidase decreases, whereas the affinity of water and lipids increases [13,54]. Therefore, different wavelengths can act through different cellular pathways based on their spectrum. Wavelengths between 905 and 980 nm can affect the pre-osteoblast cell cycle and modulate their effect in line with the therapy modality and the irradiation parameters.

However, extrapolating the most effective or dangerous parameters from a single experiment has become a challenging task, as even when data shows coherence in a single work, when collected and compared with others, the distribution is more like a patchwork than linear or hormetic [3,13,41]. Indeed, our recent evidence on the existence of window effects after irradiation of mitochondria with 980-nm laser light through progressive powers and energies could explain, in part, this specific behaviour. The 900-nm wavelengths stimulate pre-osteoblast differentiation, which is indicated by the increase in type-I collagen (Col-I) [40,44,49,53], mineralised matrix [40,44], and osteocalcin [40,44,55]. Despite this, the pulsed irradiation mode would activate cellular pathways involving transforming growth factor (TGF) and bone morphogenetic protein (BMP) [44,48,53], while the continuous modality would act on the Wnt-signalling and (Smads) 2/3-β-catenin signalling cascades [40]. Focusing on our experimental setup, literature displayed the effectiveness of 900-nm wavelengths on the proliferation process [40,43,46,50] and on cell viability [40,44,48,50], which in particular cases can, however, decrease [48,52] or be unaffected [45,46,49,52]. Nevertheless, the cellular pathway involved is only in part understood. Kunimatsu and collaborators [45] claimed that there is ERK 1/2 involvement, supported by an increase in ATP production and DNA synthesis, while the p38 mitogen-activated protein kinase (MAPK) pathway was not influenced by the 910-nm wavelength at the described parameters. Moreover, Migliario et al. [46] showed an increase in reactive oxygen species (ROS) after 980-nm irradiation, which influenced the cellular proliferation rate.

Previous data showed that 980-nm irradiation (55 J/cm^2^, 0.9 W, 0.9 W/cm^2^, 60 s) and a single energy 55 J in CW improves the MC3T3-E1 pre-osteoblast viability by affecting Bcl2 and Bax signalling. In the present work, among the lowest and the highest tested power outputs, only 45 J/cm^2^, 0.75 W and 0.75 W/cm^2^ was able to stimulate pre-osteoblast proliferation and cell viability. Nonetheless, 12 J/cm^2^, 0.20 W and 27 J/cm^2^–0.45 W values did not affect the cells. Moreover, comparing the results of previous work [40] with the present study, we revealed that irradiation with 55 J/cm^2^, 0.9 W, and 0.9 W/cm^2^ for 60 s, in CW mode, exhibited greater stimulation of MC3T3-E1 cells compared to the 45 J/cm^2^ and 0.75 W used in the current study. Additionally, when the power output shifted from 55 J/cm^2^, 0.9 W to 27 J/cm^2^, 0.45 W, this provoked a reduction in cell proliferation of about 35% at 45 J/cm^2^ and 0.75 W and 100% at 27 J/cm^2^.

The c-myc protein has long been recognised as an indispensable factor for cyclin D entry into the cell cycle machinery [56,57]. Moreover, c-myc incites the expression of cyclin-dependent kinases (Cdks) and in particular stimulates cyclins A, D1, D3, E, Cdk2, and Cdk4 [58]. We observed that 45 J/cm^2^, 0.75 W and 0.75 W/cm^2^ laser treatment enhanced cell proliferation by increasing c-myc, cyclin D3 and CDK4 cytoplasmic and nuclear distribution in pre-osteoblasts. Our findings were particularly evident when cell cultures were treated with the FT beam profile delivery system.

It is well documented that the PI3K/serine-threonine kinase (Akt) signalling pathway plays a fundamental role in cell growth and survival and can be activated by various cellular stimuli [55]. Akt is the primary mediator of the PI3K-signalling cascade, regulating cell survival via the phosphorylation of multiple downstream targets, such as pro-apoptotic proteins, transcription factors, and additional protein kinase mediators [59,60]. Of interest is the PI3K/Akt pathway, which can mediate cell survival signals through the Bcl-2 family [58,61,62]. Among the Bcl-2 family proteins, Bcl-2 and Bcl-xL have been designated as pro-survival, while Bad, Bak, Bid, and Bax are widely recognised as cell death promoters [63,64]. Notably, the PI3K/Akt pathway implicates Bcl-2 and Bcl-xL as anti-apoptotic signals [65]. Indeed, activation of the PI3K/Akt pathway leads to an increase in Bcl-2 and Bcl-xL expression and a decrease in Bad and Bax expression [66,67,68]. In this context, we investigated whether the anti-apoptotic/cell survival PI3K/Akt/Bcl-2 pathway is regulated when exposed to 45 J/cm^2^, 0.75 W and 0.75 W/cm^2^ for 60 s in CW mode. The involvement of the Akt pathway after irradiation by wavelengths of 632.8–780 nm has been reviewed by de Freitas and Hamblin [69]. However, our results suggest, for the first time, that the application of the 980-nm irradiation protocol stimulates the above-mentioned intracellular signalling cascades, leading to cell survival and activation of key anti-apoptotic mediators.

Previous [40] and present results agree with a recent characterisation of the interaction between 980-nm wavelength light and the mitochondrial respiratory chain published by our team [14]. In brief, laser light induced a decrease in the ATP production when exposed to 0.1 and 0.2 W (7.7 and 15.4 J/cm^2^). Conversely, power between 0.8 and 1.1 W (61.5–84.6 J/cm^2^) led to a progressive enhancement of energy synthesis, while the intermediate values (0.3–0.7 W; 23.1–53.8 J/cm^2^) did not have as large an effect as the 1.2–1.4 W range. 

In this way, the higher ATP production induced by the higher levels [40] could better support the proliferation and differentiation of pre-osteoblast cells, while decreasing both the powers and energies would lead to a smaller increase in cellular energy, supporting proliferation and viability, but with reduced efficacy. Indeed, the differentiation process was not stimulated, as demonstrated by our results. Out of the efficacy windows, for the lowest parameters, the effect of laser on pre-osteoblast, as well as on the ATP production, disappears [14].

Lastly, according to previous work [40], the comparison between irradiation with the FT hand-piece and ST hand-piece demonstrated a significantly more evident and reproducible cell stimulation with 1×, 3×, and 5× irradiations per week for 2 consecutive weeks with the FT modality compared to ST, which was only able to stimulate the cells with the once per week irradiation protocol.

An analysis of the mechanical signalling of bone modelling and remodelling shows interesting support for the effect of photobiomodulation on osteoblasts. Indeed, as highlighted by Robling and Turner [70], two of the earliest events in mechanotransduction signalling, which occur within the first minutes of mechanical stimulus application, are an increase in both intracellular Ca^2+^ concentrations and ATP. The process seems to involve the modulation of voltage-sensitive calcium channels and calcium stores. Soon after these first signalling events take place, nitric oxide acts as a second messenger and plays a role in the response [70]. The NO release is followed by MAP-kinase signalling and ERK1/2 activation, and ultimately, the expression of bone matrix genes to promote cell proliferation and cell viability [70]. Differentiation can also be activated by the triggering of the Wnt and β-catenin pathway [70].

Therefore, the photobiomodulation event, starting from the primary target and following the proliferative and differentiative pathways shown by our previous and present data, strongly reflects the regulation of bone homeostasis by mechanical stimuli.

Additionally, the data are strongly supported by a recent collaboration of our team with Abdel Hamid and co-workers [71], which improved the bone density of extraction sockets of skeletally mature mongrel dogs through irradiation of 980-nm and 46 J/cm^2^ for 60 s in continuous wave mode for two weeks and by the hand-piece with flat-top profile.

## 4. Materials and Methods

### 4.1. Cell Culture

The MC3T3-E1 cell line (mouse calvaria pre-osteoblasts) (ATCC, LGC Standards S.r.L Milano, Italy) was utilised in the current study. Cells were grown in minimum essential medium Eagle (αMEM) (Life Technologies, Milano, Italy) supplemented with 10% heat-inactivated fetal calf serum (HI-FCS) (Life Technologies, Milano, Italy), penicillin (100 U/mL), and streptomycin (50 µg/mL) (Life Technologies, Milano, Italy). Cells were detached using 0.25% trypsin for 2 min at room temperature and plated for each experimental protocol as below specified. The culture medium was replaced every 3 days.

### 4.2. Experimental Setup: Irradiation Tools and Laser Power Output Measurements

A 980-nm diode laser device (Doctor Smile, LAMBDA Spa, Vicenza, Italy) was utilised, with the following two delivery systems: a hand-piece with an ST beam profile (Gaussian profile) and a novel hand-piece with an FT beam profile (AB 2799, Doctor Smile, LAMBDA Spa, Vicenza, Italy). The different features between the two hand-pieces were shown in our previous works [40,72].

In this previous work [40], we showed that, with the hand-pieces fixed at distances of 3 mm and 15 mm from the power meter, only the FT profile hand-piece had a constant power output, which was comparable to the contact mode.

Therefore, to irradiate the MC3T3-E1 pre-osteoblast cells at the same laser parameters, the PM160T-HP power meter (ThorLabs, Bergkirchen, Germany) was utilised to measure the actual final powers, which were 0.75, 0.45, and 0.20 W. The cells were irradiated at these powers in CW emission mode for an exposure time of 60 s (spot size 1 cm^2^), and allowed to generate a power density of 0.75, 0.45, and 0.20 W/cm^2^ and a fluence of 45, 27, and 12 J/cm^2^, respectively.

According to our previous experimental design [40], the hand-pieces (FT and the ST) were kept at a distance of 3 mm from the cells’ monolayer on a Petri dish (without the cover) filled with 2.5 mm thickness of αMEM. 

To control the thermal increase of the irradiated samples, a FLIR ONE Pro-iOS thermal camera (FLIR Systems, Inc. designs, Portland, U.S.A.; dynamic range: −20 °C/400 °C; resolution 0.1 °C) was used during irradiation.

In accordance with our previous work [40,73], the samples were irradiated through either ST hand-piece or FT hand-piece with a frequency of one, three (alternate days), and five (every day) irradiations per week for two consecutive weeks.

In short, both the FT and ST hand-piece groups were as follows: Group 1: one irradiation per week for two weeks (1× week; day 1 and day 8); Group 2: three irradiations per week for two weeks (alternate days, 3× week; days 1, 3, 5 and 8, 10, 12); Group 3: five irradiations per week for two weeks (5× week; days 1, 2, 3, 4, 5 and 8, 9, 10, 11, 12).

The control cultures were processed under identical conditions, except that the laser device was kept off at all times (0 W).

To avoid bias, the cell growth, cell irradiation, and cell analyses (see below) were performed by different operators, and both cells’ flasks and Petri dishes were maintained in a blinded manner.

### 4.3. Assessment of the Metabolic Activity of Viable Cells (MTS)

The metabolic activity of viable cells was determined by 3-(4,5-dimethylthiazol-2-yl)-5-(3-carboxymethoxyphenyl)-2-(4-sulfophenyl)-2H-tetrazolium (MTS).

The MC3T3-E1 cells were plated at a density of 5000 cells/well on 96-well culture plates until they reached 80% confluence. Then, the cells were irradiated according to the above protocol. At the end of the treatments, the cell viability was measured by the CellTiter 96(R) AQueous One Solution assay (Promega, Milano, Italy) according to the manufacturer’s instructions.

### 4.4. Hoechst Staining to Stain the Cell Nuclei

Hoechst staining was performed using a 1:800 dilution of 2 mmol/L Hoechst dye stock (Sigma Aldrich, Milano, Italy).

### 4.5. Western Blotting

The MC3T3-E1 pre-osteoblasts were plated at 5 × 10^4^ cells/well in 6-well culture plates and treated as described above. At the end of each set of treatment, the samples were processed as previously described [40]. Membranes were immunoblotted in blocking buffer with specific antibodies: rabbit anti-runt-related transcription factor 2 (Runx-2), rabbit anti-B-cell lymphoma 2 (Bcl2), rabbit anti-B-cell lymphoma extra-large (Bclxl), and rabbit anti-osterix (Osx) (all diluted 1:400; Santa Cruz Biotechnology, DBA, Milano, Italy), rabbit anti-phosphoinositide 3-kinase/phosphorylated (p.PI3K), rabbit anti-phosphoinositide 3-kinase (PI3K), rabbit polyclonal anti-phosphate/protein kinase B (p.AKt), protein kinase B (AKt), and rabbit polyclonal anti-phosphate/extracellular signal-regulated kinases (p.ERK) (all diluted 1:800; Cell Signalling, EuroClone, Milano, Italy).

The immunoreactive bands were visualised with luminol reagents and Hyperfilm-enhanced chemiluminescence film (Euroclone, Milano, Italy) according to the manufacturer’s instructions. To normalise the bands, filters were stripped and re-probed with a mouse anti-α-tubulin antibody (Sigma-Aldrich, Milano, Italy). The band density was densitometrically quantified using NIH image (National Institute of Hearth, Rockville Pike, Bethesda MD, USA).

### 4.6. Fluorescein-FragELTM DNA Fragmentation Detection

The Fluorescein-FragELTM Kit (Calbiochem, Milano, Italy) is a method for labelling DNA breaks in apoptotic nuclei in cell preparations fixed on coverslips. Briefly, MC3T3-E1 osteoblasts were plated at 3500 cells/cm^2^ in 6-well culture dishes containing coverslips, previously cleaned and sterilised, and grown for 4 days to 80% confluence. Then, the next steps were performed as previously described [74]. Quantitative analysis was performed by direct counting under the microscope. Using the 20× objective lens, the number of apoptotic cells was counted in 30 areas (≈15 cells/area) for each slide.

### 4.7. Single Immunolabeling

MC3T3-E1 osteoblasts were plated at 3500 cells/cm^2^ in 6-well culture dishes containing coverslips, previously cleaned and sterilised, and grown for 4 days to 80% confluence. At the end of each set of irradiations, the untreated and irradiated pre-osteoblasts were fixed in 4% paraformaldehyde (PFA) and permeabilised with 0.3% Triton X-100 as previously described [75]. The cultures were then incubated for 2 h at room temperature with the following primary antibodies: rabbit anti-c-myc antibody (1:100 dilution, Cell Signaling, Euroclone, Milano, Italy), rabbit anti-cyclin D3 antibody and rabbit anti-cyclin dependent kinase (CDK4) antibody (1:50 dilution, Santa Cruz Biotechnology, DBA, Milano, Italy). After washing, cells were incubated with Alexa Fluor-488 chicken anti-rabbit IgG or with Alexa Fluor 594 goat anti-rabbit IgG (1:100 dilution, Life Technologies, Monza, Italy) for 1 h at room temperature. The reaction controls were performed by complexing the primary antibody with a relative blocking peptide or by omitting the primary antibody. Coverslips were mounted on slides with phosphate-buffered saline (PBS)/glycerol (1:1). The slides were imaged using a fluorescent microscope and fluorescence analysis was performed using a Tecan Infinite fluorimeter with a 590 nm excitor filter and emission at 635 nm for Alexa Fluor 594 or 485, and of 535 nm for Alexa Fluor 488. 

### 4.8. Statistical Analysis

Where applicable, the difference between the two groups was evaluated with an unpaired, two-tailed Student’s *t*-test using Microsoft Excel. All data were expressed as a mean ± standard error (S.E.). Values of *p* < 0.05 were considered significant. The results were representative of those acquired by independent experiments, which were repeated at least three times.

### 4.9. Literature Selection Process

To support our data and better compare it with the evidence on the effect of 900-nm wavelengths on pre-osteoblast and osteoblast cell homeostasis, we revised literature in compliance with the four-phase flow diagram shown in Supplemental Material, Figure S1. Papers were independently searched by three authors (A.D, A.A, and M.G.S.) using the PubMed, Scopus, Cochrane, and Scholar databases. The following keywords were applied to meet the strategy of the investigation: “low-level laser therapy” OR “photobiomodulation” OR “laser phototherapy” AND “osteoblast“ OR “pre-osteoblast”. Additional studies could be also identified from the references. Articles were listed and duplicates were deleted by A.D, A.A, and M.G.S. We also initially screened the works by title and abstract according to the inclusion criteria described in Figure S1. As shown in Figure S1, as only one paper complied with the criteria of selection, more inclusive criteria were applied; the mandatory evaluation of energy and the temperature was not taken into account. In vivo research, reviews, commentaries, conference abstracts, and patents as well as research on stem cells or with laser parameters that did not follow the criteria of PBM were excluded. 

## 5. Conclusions

The 900-nm wavelengths can photobiomodulate the osteoblast cell, regulating its fate according to the parameter irradiated. Our previous and present data consistently support the window effect at 980 nm, also described in extracted mitochondria [14], through activation of signalling through the PI3K/Akt/Bcl-2 pathway and cyclin family and the Wnt and Smads 2/3-β-catenin pathways.

## Data Availability

Data available on request from the authors.

## Supplementary Materials

The following are available online at https://www.mdpi.com/article/10.3390/ijms22147586/s1, Figure S1: Flow chart demonstrating the selection process. Figure S2. Pre- and osteoblast pathways modulated by 900-nm wavelengths: primary targets and secondary effects. (A) The primary target can be identified in the cytochromes of the mitochondrial respiratory chain, the nitrosothiol compounds, the bounded-water and the lipids, which after the interaction with near-infrared light, modify their energetic and vibrational state, supporting the release of adenosine triphosphate (ATP) and reactive oxygen species (ROS), nitric oxide (NO) as well as calcium (Ca2+) messengers. (B) The messengers can modulate the cellular cascades such as Wnt, TGF and BMP, which enhance cell differentiation (C) and proliferation (C’). Mesenchymal stem cell differentiates in osteoblast that in turn may evolve in osteocytes, in bone-lining cells, or die. Osteocytes are also able to stimulate the formation of osteoclasts from the stem cells (C). The number into the square bracket indicates the reference supporting the data design, while in green are showed the cellular proliferative pathway described by the experimental set-up of the present work. Image created with BioRender.com Table S1. Included studies. In vitro studies on photobiomodulation and pre- and osteoblast cells, selected after inclusion and exclusion criteria.
