# Supplementary material for: Photobiomodulation by Near-Infrared 980-nm Wavelengths Regulates Pre-Osteoblast Proliferation and Viability through the PI3K/Akt/Bcl-2 Pathway
Source: Int J Mol Sci. 2021 Jul 15;22(14):7586. doi: 10.3390/ijms22147586 (PMC8304212; doi:10.3390/ijms22147586)
Supplement: Supplementary file 1 [file ijms-22-07586-s001.zip › ijms-1237315-supplementary.pdf]

Table S1. Included studies. *In vitro* studies on photobiomodulation and pre- and osteoblast cells, selected after inclusion and exclusion criteria

Art.	Cell's type	Laser Parameters	Therapy Irradiation Mode	Methods	Results
[40]	MC3T3-E1 cell line	980-nm; 0.9W; 0.9W/cm^2, 55 J/cm^2, 55 J; 60 s. Spot-size area= 1 cm^2; irradiation mode= CW	once per week or three times per week (alternate day) or daily, five times per week. The laser was fixed 3 mm from the cell layer	***Proliferation:*** BrdU assay ***Viability:*** MTS ***ALP and Alizarin Red S histological staining, Double Immunolabeling for Osteocalcin and Collagen Type I*** ***Protein presence:*** Bcl2, Bax, Osx, Dlx5, β-catenin Smads 2/3, TGFβ	***Proliferation:*** increased ***Viability:*** increased ***ALP and Alizarin Red S histological staining:*** increased, ***Double Immunolabeling for Osteocalcin and Collagen Type I:*** increased, ***Protein presence:*** Bcl2, Bax, Osx, Dlx5, β-catenin Smads 2/3, TGFβ, increased after irradiation but only in some doses.
[43]	P.C.C of osteoblast from rat calvaria	905-nm; 0.015W, 0.008 W/cm^2, Spot-size area= 1.9 cm^2, irradiation mode= CW and 1.25 J/cm^2, 2.4J, 150 sec or 3.75 J/cm^2, 7.1J, 450 sec or 6.25 J/cm^2, 11.8J, 750 sec	One irradiation. Mode: 10 mm above the basal surface of 24-well plates	***Proliferation:*** BrdU; ***Cell Cycle Analysis; mineralized matrix:*** von Kossa technique; ***ALP activity:*** naphthol AS-BI phosphate; ***protein expression:*** Runx-2	***Proliferation:*** only 3.75 J/cm^2 affected; 1 irradiation decrease and 3 irradiations increase it; ***Cell Cycle Analysis:*** G2/M phase was induced; ***mineralized matrix:*** only 3.75 J/cm^2 affected; ***ALP activity:*** 1.25 J/cm^2 and 3.75 J/cm^2 increased ***protein expression:*** Runx2 expression increased
[44]	MG-63 cell line (human osteoblast-like cell)	904–910 nm; superpulsed 200 nsec, min-peak power 33W, average out power 0.2mW, frequency 30kHz, 5 min, tot-energy 60J for each well, 6.7 J/cm^2, Spot-size area= 9 cm^2, irradiation mode= superpulsed	Daily for 5 days then, every two days for 15 days Mode: no specified	***Viability and proliferation:*** TB exclusion test and Burker chamber; ***mineralized matrix:*** von Kossa technique; ***protein expression:*** ALP, BMP-4, BMP-7, OCN, PPARα, PPARγ, TGF-β2, COL-I	***Viability and proliferation:*** no-effect or decrement; ***mineralized matrix:*** increase; ***protein expression:*** ALP increase, BMP-4 increase, BMP-7 increase, OCN increase, PPARα increase, PPARγ increase, TGF-b2 increase, COL-I increase
[45]	MC3T3-E1 cell line	910-nm; average power 0.3W, peak-power 45W, 0.09W/cm^2, 0, 1.42, 2.85, 5.7, or 17.1 J/cm^2; pulse-rate 30kHz; pulse-duration 200ns; overall duty cycle of 0.6%, Spot-size area= 3.3 cm^2, irradiation mode= pulsed	One irradiation. The laser was fixed at a height of 35 mm from the well-plate	***DNA synthesis:*** BrdU; ***ATP synthesis:*** CellTiter-Glo™ Luminescent Cell Viability Assay; ***cell migration:*** wound makers TM® (ESSEN BIOSCIENCE); ***protein expression:*** MAPK/(ERK) 1/2, p38 MAPK, SAPK/JNK	***DNA synthesis:*** treatment with irradiation at a dose of 2.85, 5.7, or 17.1 J/cm^2 increased it; ***ATP synthesis:*** 2.85, 5.7, or 17.1 J/cm^2 significantly increased ATP synthesis; ***cell migration:*** 2.85 J/cm^2 increased; ***protein expression:*** 2.85 J/cm^2 increased ERK1/2; No changes were observed for p38 MAPK or SAPK/JNK
[46]	MC3T3-E1 cell line	980-nm; 1.57, 7.87, 15.74, 39.37, 78.75 J/cm^2 and 1, 5, 10, 25, 50 J 1, 5, 10, 25 and 50 s. irradiation mode= CW	Daily for 3 days The laser was fixed 10 cm from the cell layer	***Proliferation:*** toluidine blue solution ***Ros generation:*** H2DCFDA assay	***Proliferation:*** Irradiation increased it, with the exclusion of 1 J/cm^2. ***Ros generation:*** all irradiation increased it
[47]	P.C.C of osteoblast (human mandibular cortical bone)	915-nm; 1.5W, 0.12 W/cm^2 and 41.7, 125 or 375 s, 62.55J, 187.5J or 562.5J, 5, 15 or 45 J/cm^2, Spot-size area= 12.56 cm^2, irradiation mode= CW / 1.5W, 1.25 W/cm^2 and 4, 12 or 36 s, 5, 15 or 45 J/cm^2, Spot-size area= 1.13 cm^2; irradiation mode= CW	Daily for 3 and 6 days. Contact mode	***Proliferation:*** Coulter counter; ***mineralized matrix:*** red alizarin	***Proliferation:*** no effect; ***mineralised matrix:*** no significant differences were observed after irradiation with 1.25 W/cm^2 and fluences of 5, 15 and 45 J/cm^2. Mineralisation is higher in samples treated with 5 J/cm^2 and 0.12 W/cm^2 compared to controls.
[48]	Saos-2 cell line (human osteoblast-like cells)	915-nm; set-power 1W, output power 0.575 W, 0.3 W/cm^2 and 1, 5, 10, 20, and 50 J⁄cm^2 and 10, 48, 96, 193, and 482 s, 100 Hz pulse irradiation duty cycle of 50%, Spot-size area= 1.91 cm^2; irradiation mode= pulsed	One irradiation. The laser was fixed at a height of 19 mm from the well-plate	***Viability:*** AlamarBlue solution; ***Cell Morphology:*** LIVE/DEAD® Viability/Cytotoxicity Kit; ***DNA Quantification:*** PicoGreen®; ***cell damage: LDH, OPG, RANKL, and VEGF activity released:*** cytotoxicity detection kit	***Viability:*** 10 J/cm^2 increased it, but 20 and 50 J/cm^2 decreased. Other fluences had no-effect; ***Cell Morphology:*** 50 J⁄cm^2 induced apoptotic cell shape; 20 J⁄cm^2 had a contradictory effect with several apoptotic cells shape. Other fluences had no-effect ***DNA Quantification:*** no-difference among samples; ***cell damage: LDH, OPG, RANKL, and VEGF activity released:*** no-effect was observed
[49]	Saos-2 cell line (human osteoblast-like cells)	915-nm; set-power 1W, output power 0.575 W, 0.3 W/cm^2 and 1, 5, 10, 20, and 50 J⁄cm^2 and 10, 48, 96, 193, and 482 s, 100 Hz pulse irradiation duty cycle of 50%, Spot-size area= 1.91 cm^2; irradiation mode= pulsed	One irradiation. The laser was fixed at a height of 19 mm from the well-plate	***Viability:*** Alamar Blue solution; ***DNA Quantification:*** PicoGreen®; ***protein expression:*** COL-IA1, TGF-β1, IL-1β, MMP1; ***Supernatant Enzyme-Linked Immunosorbent Assay Measurements:*** COL-I, TGF-β1, and PGE2 Enzyme-linked.	***Viability:*** no-difference were observed; ***DNA Quantification:*** no-difference were observed; ***protein expression:*** COL-IA1 increased in all laser-irradiated groups; TGFβ1 was increased only with 10 J⁄cm^2 at 24h, but decreased at 72h; IL1β and MMP1 were not affected; ***Supernatant Enzyme-Linked Immunosorbent Assay Measurements:*** COL I, TGFβ, PGE2 were not affected

[50]	hFOB cell line (human fetal osteoblast)	940-nm; 1) 0.1 W; 0.25 W/cm^2, 22.92, 45.85 or 91.79 J⁄cm^2. 2) 0.2W, 0.5W/cm^2, 22.92, 45.85 or 91.79 J⁄cm^2. 3) 0.3W; 0.75W/cm^2; 68.78 or 137.57, 270 J/cm^2. Irradiations were performed for 90, 180 or 360 sec. Spot-size area= 0.4 cm^2; irradiation mode= CW	Daily for 7 days. The laser was fixed 14 mm above the cell layer	***Viability:*** MTT; ***Alkaline Phosphatase Activity:*** ALP Activity Colorimetric Assay; ***Osteocalcin Activity Assay:*** OCN Activity Assay Kit	***Viability:*** increase in all groups; ***Alkaline Phosphatase Activity, Osteocalcin Activity Assay***: only 0.1 W and 0.2 W irradiated for 360 s increased it.
[51]	MG-63 cell line (human osteoblast-like cell)	940-nm; power output of 70 mW, 1 W, 1W/cm^2, 3 J (14.7 s) or 1.5 W/cm^2, 3 J-(10.6 s), 4 J (19.31 s) or 1.5 W/cm^2, 4 J (12.96 s). Spot-size area= 0.126 cm^2; irradiation mode= pulsed	One irradiation. The laser was fixed 1 cm from the cell layer	***Alkaline phosphatase activity:*** colourimetric assay ***Phagocytic activity:*** carboxylated FICT-labeled latex ***Protein presence:*** monoclonal antibodies (MAbs) CD54, CD80, CD86, and HLA-DR (CD54/IOL1b, CD80, CD86, and OKDR	***Alkaline phosphatase activity:*** Incremented activity only with 1W/cm^2; ***Phagocytic activity:*** slighted reduction in phagocytic activity; ***Protein presence:*** CD54, CD86, and HLA-DR were reduced, no effects were observed on CD80 antigens.
[52]	MC3T3-E1 cell line	940-nm; 0, 0.001, 0.005, 0.008W and 5.2, 0, 0.00167, 0,00833, 0.0125 W⁄cm^2 and 0, 1, 5, and 7.5 J⁄cm^2, 600s. Spot-size area= 0.63 cm^2; irradiation mode= CW	One irradiation. Distance from the cell was not indicated	***Viability and proliferation:*** LIVE/DEAD Cell Imaging Kits - Click-iT Plus EdU Alexa Fluor 594 Imaging Kit	***Viability:*** *1 and* 5 J⁄cm^2 did not affect. 7.5 J⁄cm^2 decreased it. ***Proliferation:*** only 1 J⁄cm^2 affected it.
[53]	MG-63 cell line (human osteoblast-like cell)	940-nm; cycles of 20 ms, pulse interval/20 ms pulse length, 1 W-3 J; 1 W-4 J; 1.5 W-3 J; 1.5W-4 J Spot-size area= 1.93 cm^2; irradiation mode= pulsed.	One irradiation. The laser was fixed 1 cm from the cell layer	***Protein expression:*** Runx-2, OSX, OSC, OPG, Col-I, ALP, BMP-2, BMP-7, TGF-b1, TGF-b R1, TGF-b R2, and TGF-b R3, primers	***Protein expression:*** Irradiation increased Runx-2, ALP, Col-I, and OSX as a function of the dose but did not affect OPG or OSC gene expression. TGF-b1 was significantly higher after irradiation with 1 W/3 J; 1.5 W/3 J; 1 W/4 J; 1.5 W/4 J. BMP-2 was increased after irradiation with 1 W/3 J; 1.5 W/3 J; 1 W/4 J. No changes of BMP-7 expression were observed

ALP= Alkaline Phosphatase, ATP= adenosine triphosphate; Bax= Bcl-2-associated X protein; Bcl2= B-cell lymphoma 2; BMP= bone morphogenetic protein, BrdU= Bromodeoxyuridine, CD= cluster of differentiation; Col-I= type-I collagen; Dlx5= distal-less homeobox 5, peroxidedependent oxidation of 2'-7'; H2DCFDA= dichlorodihydrofluorescein diacetate; HLA-DR= human leukocyte antigen DR; LDH= Lactate dehydrogenase; MAPK/(ERK) ½= mitogen-activated protein kinase/ extracellular signal-regulated protein kinase; MMP= metalloproteinases; OCN= osteocalcin; OPG= osteoprotegerin; OSX= osterix; P.C.C.= primary cell culture; PPARα= peroxisome proliferators-activated receptor a; PPARγ= peroxisome; PGE2= prostaglandin E synthase; RANKL= Receptor activator of NF-kappa-B ligand; Runx-2= runt-related transcription factor; SAPK/JNK= protein kinase/Jun-amino-terminal kinase; SMADS= Small-mothers against decapentaplegic; TGF= transforming growth factor; VEGF= vascular endothelial growth factor.

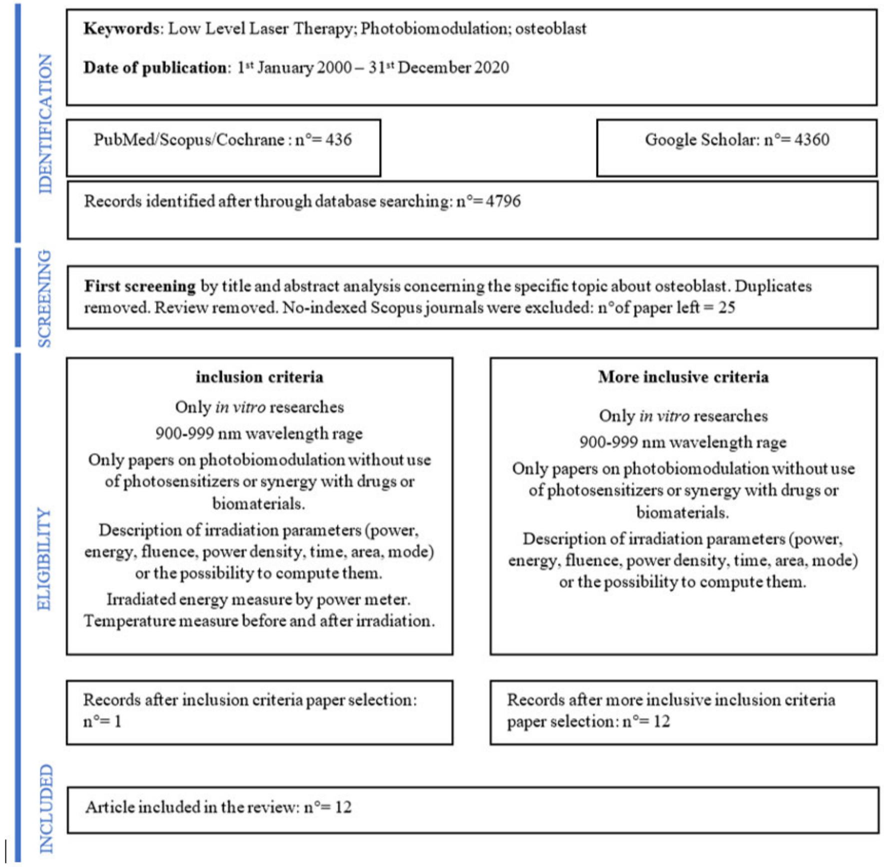

Figure S1: Flow chart demonstrating the selection process.

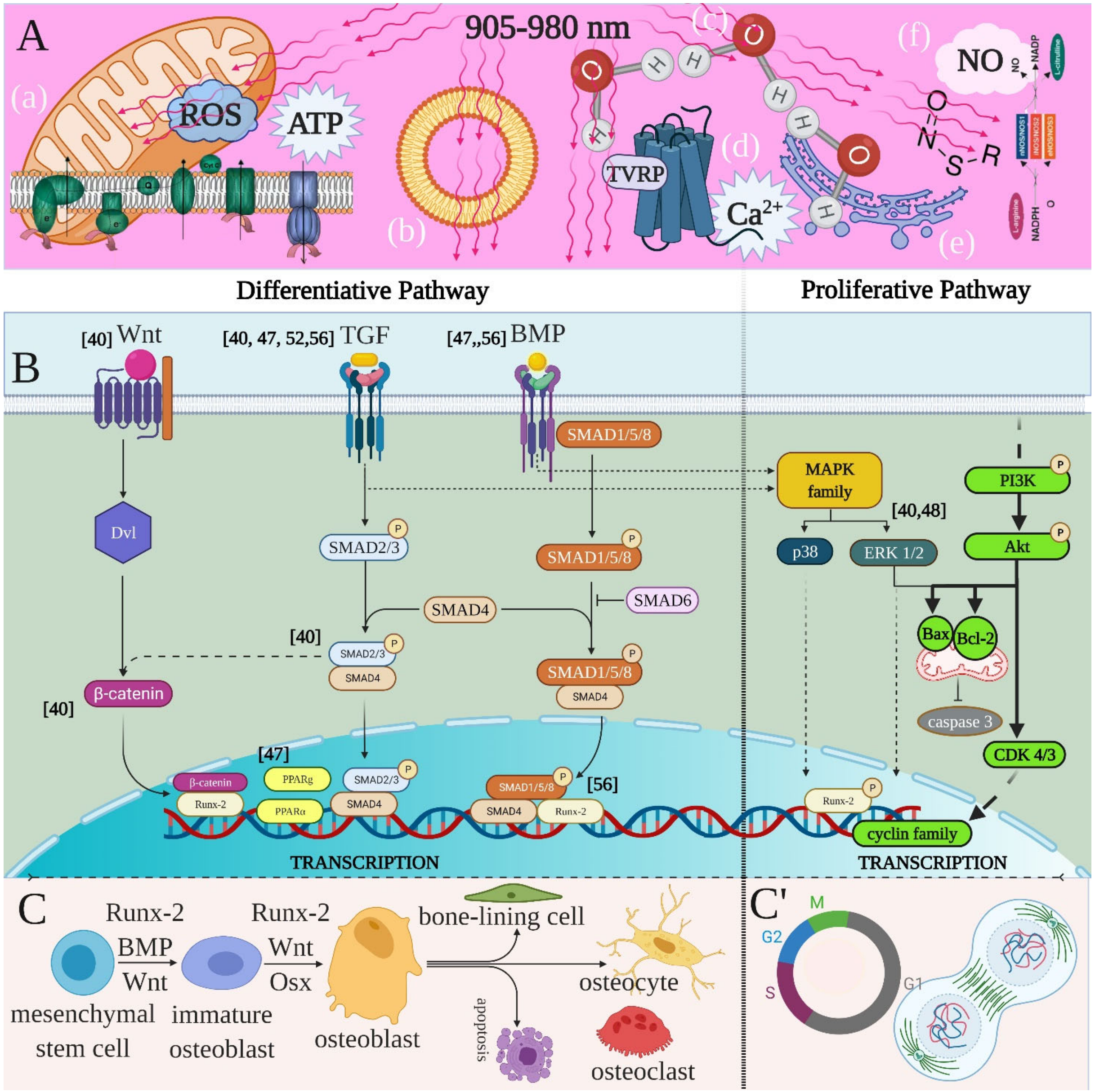
A
905-980 nm
(a)
ROS
ATP
(b)
(c)
TVRP
(d)
Ca2+
(e)
(f)
NO
Differentiative Pathway
Proliferative Pathway
B
[40] Wnt
[40, 47, 52,56] TGF
[47,,56] BMP
SMAD1/5/8
MAPK family
PI3K
Akt
[40,48]
p38
ERK 1/2
Dvl
SMAD2/3
SMAD1/5/8
SMAD4
SMAD6
Bax
Bcl-2
[40]
SMAD2/3
SMAD4
SMAD1/5/8
SMAD4
caspase 3
β-catenin
[40]
CDK 4/3
[47]
β-catenin
Runx-2
PPARg
PPARα
SMAD2/3
SMAD4
SMAD1/5/8
SMAD4
Runx-2
[56]
Runx-2
cyclin family
TRANSCRIPTION
TRANSCRIPTION
C
Runx-2
BMP
Wnt
Runx-2
Wnt
Osx
bone-lining cell
osteocyte
mesenchymal stem cell
immature osteoblast
osteoblast
apoptosis
osteoclast
C'
M
G2
S
G1

Figure S2. Pre- and osteoblast pathways modulated by 900-nm wavelengths: primary targets and secondary effects. (A) The primary target can be identified in the cytochromes of the mitochondrial respiratory chain, the nitrosothiol compounds, the bounded-water and the lipids, which after the interaction with near-infrared light, modify their energetic and vibrational state, supporting the release of adenosine triphosphate (ATP) and reactive oxygen species (ROS), nitric oxide (NO) as well as calcium (Ca2+) messengers. (B) The messengers can modulate the cellular cascades such as Wnt, TGF and BMP, which enhance cell differentiation (C) and proliferation (C'). Mesenchymal stem cell differentiates in osteoblast that in turn may evolve in osteocytes, in bone-lining cells, or die. Osteocytes are also able to stimulate the formation of osteoclasts from the stem cells (C). The number into the square bracket indicates the reference supporting the data design, while in green are showed the cellular proliferative pathway described by the experimental set-up of the present work. Image created with BioRender.com
